# Comparative Analysis of Whole-Genome Sequences of Influenza A(H1N1)pdm09 Viruses Isolated from Hospitalized and Nonhospitalized Patients Identifies Missense Mutations That Might Be Associated with Patient Hospital Admissions in Finland during 2009 to 2014

**DOI:** 10.1128/genomeA.00676-15

**Published:** 2015-07-30

**Authors:** Polina Mishel, Teija Ojala, Christian Benner, Triin Lakspere, Dmitrii Bychkov, Petri Jalovaara, Laura Kakkola, Hannimari Kallio-Kokko, Anu Kantele, Matti Kankainen, Niina Ikonen, Samuli Ripatti, Ilkka Julkunen, Denis E. Kainov

**Affiliations:** a The Institute for Molecular Medicine Finland (FIMM), University of Helsinki, Helsinki, Finland; b Institute of Biomedicine, Pharmacology, University of Helsinki, Helsinki, Finland; c Department of Virology, University of Turku, Turku, Finland; d Helsinki University Hospital Laboratory (HUSLAB), Helsinki, Finland; e Department of Medicine, University of Helsinki, Helsinki, Finland; f Inflammation Center, Helsinki University Hospital, Helsinki, Finland; g National Institute for Health and Welfare (THL), Helsinki, Finland; h Department of Public Health, University of Helsinki, Helsinki, Finland; i Wellcome Trust Sanger Institute, Cambridge, United Kingdom

## Abstract

Here, we report 40 new whole-genome sequences of influenza A(H1N1)pdm09 viruses isolated from Finnish patients during 2009 to 2014. A preliminary analysis of these and 186 other whole genomes of influenza A(H1N1)pdm09 viruses isolated from hospitalized and nonhospitalized patients during 2009 to 2014 in Finland revealed several viral mutations that might be associated with patient hospitalizations.

## GENOME ANNOUNCEMENT

Human influenza A viruses cause epidemics and pandemics (http://www.who.int/topics/influenza/en/). The most recent influenza pandemic and the epidemics following it were associated with influenza A(H1N1)pdm09 viruses. Infections with these viruses in most cases were self-limiting and led to the recovery of infected individuals within 1 week. However, in some cases, infections with A(H1N1)pdm09 viruses resulted in hospitalizations. Many different factors might be associated with the hospitalization of patients infected with A(H1N1)pdm09 viruses, including viral and host genetics, underlying health conditions, and lifestyle and environmental factors.

In this study, we searched for viral genetic factors that might be associated with patient hospitalizations. In particular, we sequenced the whole genomes of 40 influenza A(H1N1)pdm09 strains isolated from nonhospitalized and hospitalized patients (Finland, 2009 to 2014) who were diagnosed with influenza A virus infection. The sequencing procedure is described elsewhere (1). We analyzed the resulting sequences together with 186 other sequences of influenza strains isolated during the same period in Finland (1–4). A comparative analysis of whole-genome sequences of influenza strains isolated from 56 hospitalized and 170 nonhospitalized Finnish patients revealed 29 missense mutations that might be associated with patient hospitalizations (*P* < 0.00005, chi-square test of independence). Ten mutations were found in viral hemagglutinin (HA) (D114N, S202T, E391K, S468N and E516K; numbering is based on the mature HA protein with the signal sequence) and neuraminidase (NA) (N44S, I106V, N200S, V241I, and N369K) glycoproteins. Thirteen signatures were found in polymerase subunits polymerase acidic (PA) (N321K), polymerase basic 1 (PB1) (G154D, I397M, I435T, and V525I), and PB2 (R54K, M66I, D195N, R293K, N340K, V344M, I354L, and V731I). Two signatures were found in matrix 1 (M1) (V80I and K230I), one in M2 (D21G), one in nonstructural 1 (NS1) (L90I), one in nucleoprotein (NP) (S498N), and one in PA-X (R221Q). No signatures were found in NS2/NEP.

Interestingly, 12 of the 29 mutations were previously identified. In particular, mutations at positions 114/97, 202/185, 391/374, and 468/451 of HA (numbering is based on the mature HA protein with/without the signal sequence), 106, 241, and 369 of NA, 321 of PA, 344 and 354 of PB2, 80 of M1, and their combinations were shown to enhance virus replication in human cells and mice and contribute to an increased severity of A(H1N1)pdm09 virus infections in patients admitted to hospitals in England during 2009 to 2011 (5). Mutations at positions 340 of PB2 and 106 of NA were associated previously with influenza virus virulence in humans and mice (6–8). We were unable to detect the D222G mutation in HA (numbering is based on the mature HA protein lacking the signal sequence), which was proposed to contribute to the severity of the disease (9). In contrast, we observed that this mutation (N/D239G in full-length HA with signal peptide) was associated with virus propagation in MDCK cell culture (9). Thus, we validated 12 known and identified 17 novel viral factors that are potentially associated with patient hospitalization.

We found that the viruses that circulated in 2013/2014 in Finland drifted from the earliest Finnish A(H1N1)pdm09 isolate (A/Helsinki/P15/2009), for which the whole-genome sequence is available, by ca. 1.3% at the amino acid level. This indicates that novel mutations associated with patient hospitalizations may emerge and should therefore be monitored.

### Nucleotide sequence accession numbers.

The full-genome sequences of influenza A viruses have been deposited in GenBank under the accession numbers KM437818 to KM437825, KM437826 to KM437833, KM437770 to KM437777, KM437778 to KM437785, KM437706 to KM437713, KM437842 to KM437849, KM437786 to KM437793, KM437794 to KM437801, KM437682 to KM437689, KM437658 to KM437665, KM437666 to KM437673, KM437674 to KM437681, KM437690 to KM437697, KM437698 to KM437705, KM437722 to KM437729, KM366559 to KM366566, KM366351 to KM366358, KM366359 to KM366366, KM366367 to KM366374, KM366391 to KM366398, KM366399 to KM366406, KM366407 to KM366414, KM366415 to KM366422, KM366423 to KM366430, KM366431 to KM366438, KM366439 to KM366446, KM366447 to KM366454, KM366463 to KM366470, KM366479 to KM366486, KM366487 to KM366494, KM366495 to KM366502, KM366503 to KM366510, KM366511 to KM366518, KM366519 to KM366526, KM366527 to KM366534, KM366543 to KM366550, KM366551 to KM366558, KM366567 to KM366574, KM366575 to KM366582, and KM366383 to KM366390.
